# Supplementary material for: Genomic and Phenotypic Analysis of Linezolid-Resistant Staphylococcus epidermidis in a Tertiary Hospital in Innsbruck, Austria
Source: Microorganisms. 2021 May 10;9(5):1023. doi: 10.3390/microorganisms9051023 (PMC8150687; doi:10.3390/microorganisms9051023)
Supplement: Supplementary file 1 [file microorganisms-09-01023-s001.zip › Supplementary Files.pdf]

Microorganisms

Genomic and phenotypic analysis of Linezolid-resistant *Staphylococcus epidermidis* a tertiary hospital in Innsbruck, Austria

Supplementary Material

Silke Huber[1*], Miriam A. Knoll[1*], Michael Berktold[1], Reinhard Würzner[1], Anita Brindlmayer[2], Viktoria Weber[2], Andreas E. Posch[3], Katharina Mrazek[3], Sarah Lepuschitz[3], Michael Ante[3], Stephan Beisken[3], Dorothea Orth-Höller[4#], Johannes Weinberger[3]

*authors contributed equally

#corresponding author

[1]Institute of Hygiene and Medical Microbiology, Medical University of Innsbruck, Innsbruck, Austria

[2]Center for Biomedical Technology, Department for Biomedical Research, Danube University Krems, Krems, Austria

[3]Ares Genetics GmbH, Vienna, Austria

[4]MB-LAB – Clinical Microbiology Laboratory, Innsbruck, Austria

Correspondence to:

Dorothea Orth-Höller, MD

MB-LAB Clinical Microbiology Laboratory

Franz-Fischer-Straße 7b

6020 Innsbruck, Austria

Email: do@mb-lab.com

Phone: +43 (0)512 563380

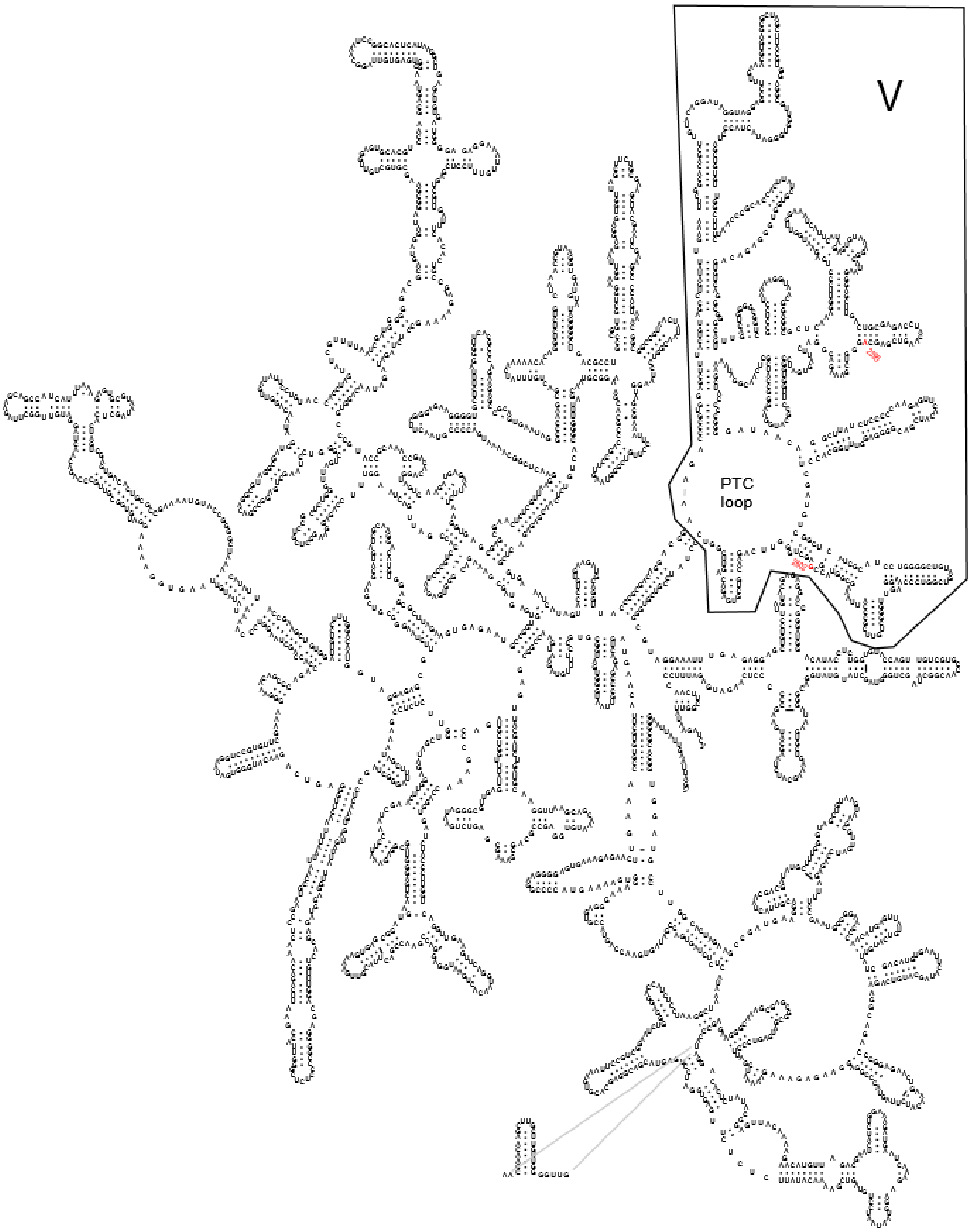

Figure S1. Novel mutations in the 23S rRNA region linked to linezolid resistance in *S. epidermidis*. Novel variants associated with linezolid resistance include A131G, G262U, G1256U, G1273A, and A2395AG. Interestingly, only insertion A2395AG maps into Domain V. The image was obtained from RNAcentral, domain V, target peptidyltransferase centre (PTC) loop, and mutations within the domain V were highlighted according to the RNAcentral Consortium.

Table S1. Overview of point mutations of the 232S rRNA and ribosomal protein L3 and L4 that were investigated.

Investigated point mutations associated with linezolid resistance	
Mutation of 23S rRNA (*E. coli* numbering)	A2062C, A2453C, A2453G, A2503G, A2503U, A2572U, C2452U, C2499U, C2534U, C2571G, C2612A, G2032A, G2032C, G2032U, G2061U, G2447U, G2505A, G2528U, G2576U, G2603U, G2608C, G2608U, U2132A, U2211G, U2357A, U2500A, U2500C, U2504A, U2504C, U2504G
Mutation of 23S rRNA (*S.aureus* numbering)	G2603U
Mutation of the ribosomal protein L3	V96D, Q136L, G137D, G137S, G139R, H146P, H146R, F147I, F147L, F147Y, P151L, G152D, V154L, G155R, M156T, A157R, S158F, D159E, D159Y, M169L
Mutation of the ribosomal protein L4	N20S, A22T, K68Q, G71D, L108S, A133T, V155I

Table S2. Number of valid data for general, laboratory and clinical parameters collected. Percentages are given in brackets.

Parameters	Numbers and percentage of valid values
General parameters	
Date of sampling	130 (100.00)
Gender	130 (100.00)
Age	130 (100.00)
Weight	112 (86.15)
Laboratory parameters	
ASAT	128 (98.46)
ALAT	128 (98.46)
γGT	128 (98.46)
CRP	129 (99.23)
PCT	60 (46.15)*
Clinical parameters	
Ward	130 (100.00)
Duration of hospitalization	130 (100.00)
Source of Isolate	129 (99.23)
Linezolid use	108 (83.08)
Antimicrobial treatment	127 (97.69)
Death	130 (100.00)

ASAT: aspartate transaminase; ALAT: alanine transaminase; γGT: gamma-glutamyltransferase; CRP: C-reactive protein; PCT: procalcitonin
*excluded from further statistical analysis

Table S3. Results of variant calling identified potential single nucleotide polymorphisms (SNPs) within 23S rRNA mutations causing resistance to linezolid in *Staphylococcus epidermidis*.

SNPs	Number of respective SNPs within 12 Isolates with unknown Resistance Mechanisms	Total Number of respective SNPs within 129 Isolates
AE015929.1594723.1597555:A131G	1	9
AE015929.1594723.1597555:G2602U	0	12
AE015929.1719192.1722024:C1596U	3	27
AE015929.1719192.1722024:G1914A	0	3
AE015929.1719192.1722024:G2602U	1	86
AE015929.1808510.1811337:G262U	0	64
AE015929.1808510.1811337:G1256U	1	10
AE015929.1808510.1811337:G1273A	4	81
AE015929.1808510.1811337:G2597U	0	15
AE015929.2332749.2335580:A2395AG	12	56
AE015929.2332749.2335580:G2601U	1	90
AE015929.2377479.2380311:G267U	0	7
AE015929.2377479.2380311:G2602U	0	75
Total of 13 SNPs	23	535
